# Signaling in T cells – is anything the m(a)TOR with the picture(s)?

**DOI:** 10.12688/f1000research.7027.1

**Published:** 2016-02-18

**Authors:** Mark Boothby

**Affiliations:** 1 Department of Pathology, Microbiology & Immunology, Vanderbilt University, Nashville, TN, USA

**Keywords:** T-cell, T-cell signalling, mTOR

## Abstract

The excitement surrounding checkpoint inhibitors in the treatment of patients with cancer exemplifies a triumph of the long-term value of investing in basic science and fundamental questions of T-cell signaling. The pharmaceutical future actively embraces ways of making more patients’ cancers responsive to these inhibitors. Such a process will be aided by elucidation of signaling and regulation. With thousands of articles spread across almost 30 years, this commentary can touch only on portions of the canonical picture of T-cell signaling and provide a few parables from work on mammalian (or mechanistic) target of rapamycin (mTOR) pathways as they link to early and later phases of lymphocyte activation. The piece will turn a critical eye to some issues with models about these pathways in T cells. Many of the best insights lie in the future despite all that is uncovered already, but a contention is that further therapeutic successes will be fostered by dealing with disparities among findings and attention to the temporal, spatial, and stochastic aspects of T-cell responses. Finally, thoughts on some (though not all) items urgently needed for future progress will be mooted.

## A recap of “T-cell activation” and the checkpoint inhibitor breakthrough

Checkpoint inhibitors represent a breakthrough treatment class for patients with cancer ^1– 3^ and derive from investing in basic science and fundamental questions of T-cell signaling. What are the roots of this revolution and how does it relate to signaling inside T cells? On conceptual grounds, Bretscher and Cohn hypothesized that T-cell activation should require two signals ^4^. Myriad studies established the principle that T cells would be activated through an antigen receptor and that this T-cell antigen receptor (TCR) was stimulated by encounter with a cell whose surface bears a specialized protein encoded in the major histocompatibility complex (MHC) combined with a suitable peptide ^5, 6^. TCR interactions with an appropriate MHC-peptide complex, however, could yield a state of unresponsiveness when an arcane chemical fixative was used to analyze antigen presentation ^7^. These findings implied that TCR stimulation was not enough; a co-stimulatory molecule was required, consistent with the model by Bretscher and Cohn. A race to elucidate the mechanism culminated in identification of the CD28 co-stimulatory receptor as the protein stimulated by a fixation-sensitive ligand on the antigen-presenting cell (APC) displaying a stimulatory MHC-peptide complex ^8^. Of note, few if any of these investigations were driven by “cancer research”.

This galaxy of investigation in turn led to families of co-stimulatory (4-1BB, ICOS, etc.) and co-inhibitory receptors (among which are CTLA-4 and PD-1, each a target of one class of pharmaceutical to enhance immune activity hitting tumors) ^9– 11^. In contrast to CD28, most of these crucial collaborators in TCR signal-interpretation are induced after T-cell activation. In parallel, the discovery of these regulators of T-cell activation, proliferation, and function prompted identification of their ligands, typically proteins expressed on the surfaces of APCs. These proteins that engage the co-stimulatory and -inhibitory receptors also follow a theme dividing constitutive (for instance, on so-called “professional APCs”) and inducible expression. Inflammation, including local cytokines but perhaps also intracellular sensors of cell stress (such as inflammasomes, Nod-like receptors, or cGAS), can promote induction of the ligands (e.g. PD-L1 or PD-L2) for the co-regulators (e.g. PD-1).

However, much of the emergence of effector functions for a naïve T cell takes place days after it first starts being activated, and intra-vital imaging reveals striking journeys traveled by the motile T lymphocyte and its daughters after an activating encounter ^12– 15^. At present, sufficient breadth and depth of information about these variable and less deterministic aspects of T-cell population development are sorely lacking, in part because of limitations in the present state of technology for such analyses. Accordingly, a missing link and frontier for investigation must be the time element and dynamism of influences on the T cell even after its initial encounter with an APC bearing stimulatory peptide-MHC complexes and cell-scale resolution of the variegated nutritional environments in which T cells operate.

## Drilling in the message(s): TCR-activated signal transduction

The simple canonical outline of early signal transduction activated by TCR engagement and CD28 co-stimulation has been beautifully reviewed, most recently by Malissen ^16, 17^, who highlights points of uncertainty. In the general model, Src-family kinases initiate protein tyrosine phosphorylation at characteristic tyrosines in ITAMs (immunoreceptor tyrosine activation motifs) on the cytoplasmic tails of chains essential to the overall TCR complex. Phospho-ITAMs recruit adapter proteins crucial for signal propagation and diversification. One of these recruits is another protein tyrosine kinase, ZAP70, which phosphorylates yet further targets in the cascade. Of note, genetic and pharmacological analyses reveal ZAP70 as a prototype of a relay essential for one type of outcome from TCR engagement yet not another ^18– 20^. One adapter notable among the relays downstream from ZAP70, LAT, can interact with several distinct classes of further adapter and transducer proteins. This arrangement affords the capacity to diversify the nature of signal ^21– 24^. Elegant genetic experiments provide evidence of the essential nature of most of the proteins and even of distinct functions for specific tyrosines within their sequences ^21^. As detailed in later sections, activity of multi-protein complexes containing the target of rapamycin (mTOR) serine-threonine kinase is induced downstream from these earliest and most rapid TCR-triggered signals.

Such monomorphic descriptions skip over vital parts of the body of data that probably are as crucial for the biology and the adaptive value of the system as its basic outline. First, let us consider the naïve T cell. Here, particular cases of individual TCR and their interactions with activating ligands indicated that quantitative and qualitative differences (the densities of peptide-MHC complexes and the exact sequence of the peptide) led not only to quantitative and qualitative differences in later-phase signaling (for instance, that of ERK MAP kinases) but also to altered effector fate and function ^25– 29^. A functional reinforcement of these fundamental findings emerged in elegant experiments seeking to sketch out the constraints on probabilities of an individual TCR yielding particular effector subsets *in vivo* by use of single-progenitor transfers ^30, 31^. In short, how a TCR signals varies according to the TCR. A second layer is that, even within the single-cell transfer results, the variance in distributions for a given TCR was substantial and probably reflects stochastic events that may even have a largely random origin.

So, an item for the future is to determine how much adaptive value in immunity builds on stochastic variation in the signaling from an individual TCR and the immune analogue of Heisenberg’s uncertainty principle. As a first step on this path, methods of single-cell analysis and modeling ^32– 35^ will be an exciting frontier. Older evidence showing that the diverse populations of memory T cells arising from a given TCR signal differently from their naïve progenitor population presents a third issue. In line with the points raised earlier in this paragraph, this picture is complicated by the fact that different results were obtained depending on the TCR. Finally, another exciting point is that the functional impact of a signal or its subcellular localization can depend on whether the TCR is on a suppressive, tumor-promoting regulatory T (Treg) cell or some other form of CD4 T cell ^36^.

## PI3K-mTOR signaling and the T cell

**Figure 1.**
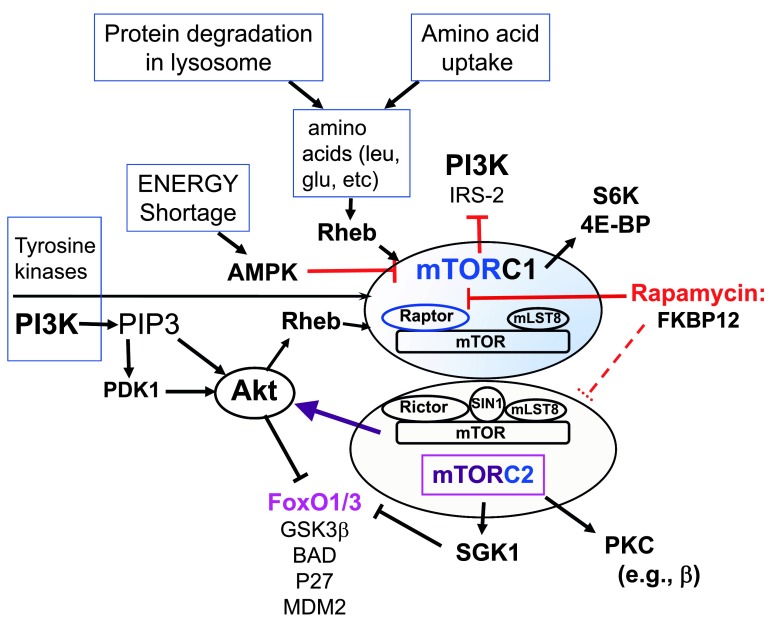
A simplified summary of the two distinct mammalian (or mechanistic) target of rapamycin (mTOR) signaling complexes, with some of the inputs (activators) and outputs. Details are outlined in the text.

The lipid kinase phosphatidylinositol 3-kinase (PI3K) is another signal transduction mechanism initiated by the TCR or the combination of TCR and co-stimulatory receptor engagement. Notably, co-stimulatory receptors (e.g. CD28 and, even more strongly, ICOS) enhance generation of the lipid product of PI3K (i.e. phosphatidylinositol 3,4,5 triphosphate, or PIP3) ^37– 42^. This in turn enhances activity of serine-threonine kinases, including PIP3-dependent kinase-1 (PDK1) and its targets AKT, protein kinases C (PKC), and SGK1, as well as mTOR ^41– 43^ ( Figure 1). Conversely, there is evidence that engagement of at least some of the co-inhibitory receptors decreases PI3K and mTOR activity ^44^ but also that they stimulate these pathways ^45^. Ultimately, these signals change gene expression profiles; alterations in DNA-binding transcription factors or other layers in the machinery for transcriptional regulation are probably a major part of the mechanism for such changes. Members of a branch of the Forkhead box transcription factor family (i.e. FoxO1 and FoxO3) are notable targets of these pathways. In particular, FoxO nuclear export and cytosolic retention are prompted by phosphorylation. The kinases for this negative regulation of FoxO are AKT – as regulated by phosphorylation by both PDK1 and mTOR in one of its two functional complexes – and SGK1 ^41, 42, 46^. SGK1, like AKT, is activated by mTORC2. Though discussed less here because of constraints on length and focus, pertinent inputs from the stimulation of cytokine receptors by the complex mixtures of their ligands in the micro-environments of T cells also impact the behavior (survival, proliferation, migration, and differentiation) of lymphocytes. In aggregate, the sentences and paragraphs formed from the rich lexicon of cytokines are sometimes distilled to consideration of “signal 3” to integrate with the graded signals of TCR (signal 1) and co-stimulation/co-inhibition (signal 2).

All together, this framework provides insights into – or “explains” – co-regulation, the various functional states of T cells, and the amount of functional activity these functional classes may retain (for instance, in the setting of anti-tumor responses of T cells). A central element of the story involves the capacity of CD4 ^+^ T cells to form various helper subsets but also both thymus-derived and peripherally generated suppressive Treg cells. Growth of a range of cancers is promoted (Treg) or restrained (cytotoxic CD8 T cells and some of the effector subsets) by subtypes of T cell ^47– 50^. Experiments pushing signaling to or beyond extremes of the dynamic range in physiology provided the result that persistently activated AKT blocked Treg fate ^51^. These findings provide the crystal nucleus for a model in which modulation of the levels of AKT and mTOR provides a means to regulate a balance between taking on the Treg “fate” and that of T helper effectors such as the Th17 cell. Such a model harmonized with evidence derived from elimination of the enzyme mTOR from T lineage cells or inactivation of mTOR complex 2 ^52, 53^; in the extreme, this created a remarkable imbalance in which the effector fate after activation of naïve CD4 ^+^ T cells was almost completely diverted into that of cells expressing the “master regulator of Treg formation”, FoxP3 ^52^.

In broader settings such as infection or cancer biology, of course, accounting for the involvement of and impact on thymus-derived Treg cells is a vital issue. Clearly, dominant suppression remains essential even after initial establishment of immune repertoires, since elimination of Treg from the mature mouse unleashed auto-inflammation ^54^. Short of killing off FoxP3-expressing cells ^54^, fate-marking experiments suggest that suppressive function is retained if FoxP3 is eliminated after establishment of the Treg state, although there is some divergence of findings and controversy in this area ^55, 56^. In a likely link to AKT, however, gene deletion analyses of the transcription factors FoxO1 and FoxO3a provide evidence that these transcription factors each regulate the formation of thymus-derived Treg cells but also that complete loss of FoxO1 undermined the functional capacity of FoxP3 ^+^ suppressors ^57– 59^. At one level, then, the collective findings yield a beautiful picture. Interference with PI3K-activating co-stimulation must decrease AKT-driven FoxO phosphorylation, thereby promoting its nuclear localization and Treg function.

Many further findings add paint to this model while also raising questions as to “who’s in charge of mTOR activity?” Most broadly, work of the past several years has increasingly highlighted that the capacity to supply amino acids to a juxta-lysosomal locale also appears crucial for activity of mTORC1 and pathways downstream from it ^60– 63^. Work with mouse systems provides evidence that G-protein-coupled receptors for complement fragments C3a and C5a are essential for Treg function and shows that most of the CD4 T cells’ mTOR activity is lost when both C3aR and C5aR are absent ^64, 65^. In parallel, a body of work with human CD4 T cells indicates that an accessory protein in the complement system, CD46, is particularly crucial for enhancing leucine uptake rates and mTORC1 activity a day after T-cell stimulation or co-stimulation by antibody cross-linking ^66^. Though not tied definitively to FoxP3, the body of studies on complement and CD46 suggest that the suppressive activity yielded after activation is influenced through this pathway ^67^. This work with human cells starts to get at one of the great gaps in the canon (i.e. the time element and an assessment of how mechanisms evolve within an individual T cell and its progeny as it moves through time and different locations). Thus, much of the evidence on signaling in T-cell activation focuses on time points that are quite early in relation to the time at which effector phenotype or an enhanced probability of memory fate arises. Work with mouse and human Treg cells emphasizes a vital role for the satiety-regulating hormone leptin in T-cell physiology along with a temporal function of leptin receptor-induced mTORC1 activity in repression of Treg proliferation ^68, 69^. This role of leptin in mTOR regulation, along with earlier evidence of its capacity to enhance interferon-gamma (IFN-γ) production from cultured CD4 T cells ^70^, is intriguing in the context of metabolism of the patient with cancer. Another important aspect of mTOR regulation is suggested from the combination of recent and older work on signaling by Notch receptors. In addition to a requirement for Notch in thymic T-cell development, a variety of articles have linked this receptor to regulation of the balance of effector cell subset formation, including evidence for Notch-stimulated mTOR controlling Treg ^71– 82^. Of note, Notch stimulates both mTORC1 and mTORC2 activity; each of these branches is important for T helper subset specification, including distinct functions of mTORC1 and mTORC2 in Treg ^53, 82– 85^, and is needed for Notch function in thymocytes ^84, 85^. PI3K-mTOR activity initiated by the inducible co-stimulator ICOS is yet another driver of mature T-cell differentiation (e.g. the follicular helper subset, TFH) ^40, 86^.

## So, what could be the m(a)TOR with the beautiful picture?

**Figure 2.**
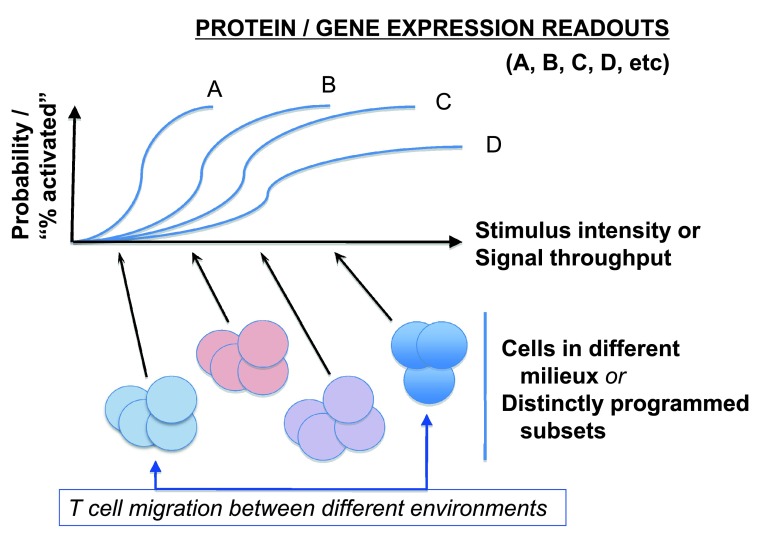
Challenges in extrapolating complete (or almost complete) and permanent loss-of-function results to sensors poised with different dose-response curves and threshold settings in biological ranges. As highlighted by evolving single-cell stimulus-response research ^34, 35^, a given stimulus will often yield a probability of evoked response well below 1, and this stimulus-probability curve will vary within a series of gene expression or downstream signal intensities pertinent to T-cell differentiation or function. Accordingly, as cell conditions or pre-existing programs vary or as different extents of impairment to a given signal relay are imposed, the responses will exhibit variegation among cells in a population. Similar principles are postulated almost certainly to apply to the time element (e.g. how long does it take for a condition to change histone post-translational modifications or epigenetic modification of DNA or to change the level of expression of a target gene?). As noted in Figure 4, local conditions vary even within a single tumor mass (setting aside known differences between metastatic and primary tumors).

Notwithstanding the beautiful picture, the PI3K-mTOR signaling pathway further exemplifies some pitfalls of relying on a binary style of drawing conclusions and the previously noted complications in conceiving of T-cell differentiation independent from the dynamism of time and space (hence, micro-environment). At present, the dominant experimental tool consists of stable, nearly complete losses of function. This suggests a need for caution about physiological perturbation-response relationships ( Figure 2). This concern is strengthened by evidence that the magnitude and duration of mTOR kinase activation vary according to the circumstances of stimulation (including the mix and timing of cytokine exposures after initial activation) and work showing that the effects of rapamycin depend on the strength and nature of stimulation ^87, 88^. The preceding section suggests yet another gap in our approaches and understanding. As ever more phenomena are attributed to the activation of mTOR or its nearly complete failure — e.g. 89, 90 — in addition to previously cited work (e.g. 39, 40, 61, 62, 65, 66, 69), it will be intriguing to sort out mechanisms; already, well over a half-dozen disparate receptors with very different ligands are each reportedly necessary for approximately 80% (or more) of the activity on a branch of the mTOR pathway.

There might be some super-complex requiring all these disparate elements at once. Also, there may prove to be subtle variations in the time course or protocols even as the nature of developing scientific stories emphasizes “optimizing conditions” to maximize a particular observation. On a mechanistic note, one potential resolution of the conundrum is whether there are relay or cross-talk effects (e.g. whether mTOR activity stimulated by one receptor depends on antecedent induction by a different stimulus). This model may apply in the case of Notch-stimulated mTOR, which then feeds into “tuning” TCR sensitivity ^73^, a model separately proposed for the transcription factor nuclear factor-kappa-B (NF-κB) in thymocytes ^91^. Another possibility links to changes in subcellular localization. Key kinases in this pathway partition among compartments (for instance, AKT and the mTORC1 target S6K) ^92– 95^. In the case of FoxO phosphorylation, it is appealing to consider that nuclear AKT is a crucial element of the equation. Of note, the efficiency of nuclear sampling for phosphorylated forms of AKT varies dramatically among means of cell extraction (an analytic problem that is even worse with intracellular staining for flow cytometry). So, there may be differential sampling of these compartments (e.g. nucleus versus cytosol) in various articles. Among the mutually compatible possibilities is that function is effected in large part via relays and supra-molecular complexes akin to signaling involving MAPK or NF-κB activation ^96– 99^.

**Figure 3.**
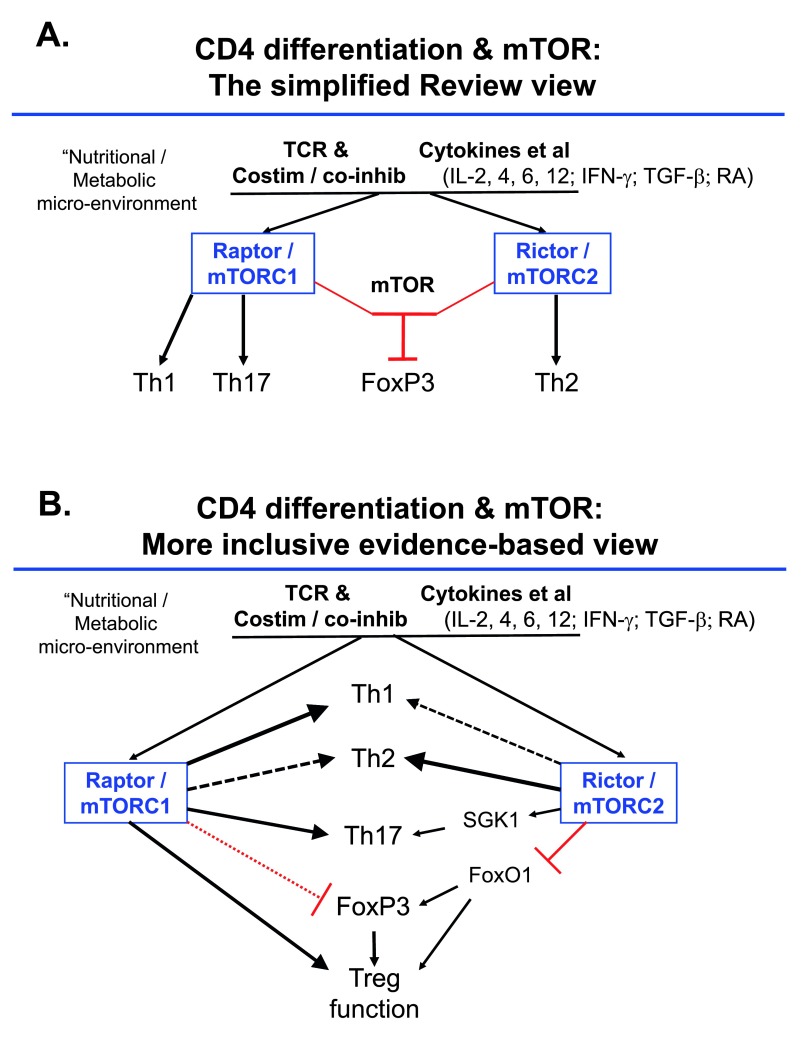
Over-simplified and more literature-based pictures of mTOR regulation of the acquired functions of mature CD4 ^+^ T-cells. ( **A**) An oversimplified view segregating functions of mammalian (or mechanistic) target of rapamycin (mTOR) complexes with distinct functions mapping one on one with T helper (Th) subsets. ( **B**) A partial integration of more complete information as represented in the cited articles ^51– 53, 57– 59, 79– 83^ and related work. Notable differences include that, presumably as a function of differences in conditions, the literature supports roles for each mTOR complex in promoting each of the peripherally acquired CD4 effector phenotypes (Th1, Th2, Th17), while the effects on regulatory T (Treg) are complex, with mTOR and mTORC2 shown to restrain induction of FoxP3 ^52, 53^ but mTORC1 vital for the suppressive function of these cells ^83^. If non-drastic decreases in nuclear FoxO1 are assumed to attenuate Treg function ^59^, then enhanced mTORC2 activity in Treg might decrease their inhibitory properties. The connection of mTORC2 to Th17 function is inferred from mTORC2 activation of SGK1 and the function of this latter factor in promoting pathogenic Th17 ^46^. Abbreviations: IFN-γ, interferon-gamma; IL, interleukin; RA, retinoic acid; TCR, T-cell receptor; TGF-β, transforming growth factor-beta.

Second, important work on FoxO1 and stability of immune homeostasis mediated by Treg used a knock-in that permitted quantitation of the extent to which TCR stimulation could evict Foxo1 from the nucleus. In this analysis, two key findings were that (i) for FoxP3 ^+^ (Treg) and FoxP3 ^−^ (Tconv) cells, signal intensities for both Erk and the mTOR–AKT–FoxO1 were quite different, as was the extent of FoxO1 redistribution, and (ii) at best, partial nuclear exclusion was driven when focusing on Treg ^59^. Accordingly, even haplo-insufficiency for FoxO1 may overestimate the extreme end of a dynamic range that is achieved *in vivo*. An intriguing side point pertaining to transforming growth factor-beta-rich tumor environments is that this cytokine activates mTOR and AKT ^100– 102^ even though AKT is supposed to suppress Treg fate or function. In light of the evidence that signal distribution and “interpretation” within Treg can differ dramatically from those in the conventional CD4 ^+^ T cell, detailed analysis of how PD-1 actually affects mTORC1, mTORC2, and other signaling pathways is needed. More broadly, there is a need in the field to seem less absolute and oversimplistic. In addition to the factors already noted, recent work in a developmental system provides strong evidence that the use of longer-term and complete gene inactivation or loss of function can elicit compensatory circuits that are avoided when a signal is attenuated more acutely with RNA interference (RNAi) ^103^. As one working example, disparate articles even within the spheres of both CTL and conventional helper T-cell differentiation observe quite different outcomes (both qualitatively and quantitatively) with perturbing the same pathway ^53, 79– 81, 83, 104– 107^ ( Figure 3). These differences probably represent combinations of fine gradations in the timing and extent of loss-of-function perturbations and hence distinct points on uncharacterized dose-response curves, along with variance among models and, who knows, maybe even microbiomes.

**Figure 4.**
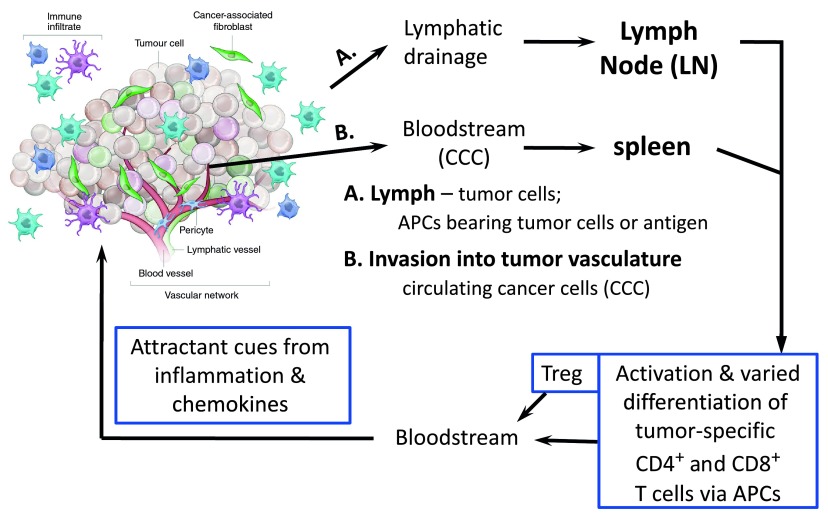
T cells and the tumor. Shown is a simplified overview of the multiple and diverse sites at which each type of T cell may encounter signals and tumor-derived antigens during growth and metastasis for carcinomas, together with a diagram underscoring likely variations of malignant cells within the tumor mass itself. In addition to action within the tumor itself, which may not be a site of initial tumor antigen presentation to and activation of T cells, either live or dead tumor cells, or antigens derived from them, traffic and flow to secondary lymphoid organs (spleen; lymph node), in either particulate or cell-associated (e.g. within a dendritic or movable phagocytic cell) form. APC, antigen-presenting cell; Treg, regulatory T.

Third, T cells are quite the moving target. Intra-vital imaging, albeit with caveats relating to the potential impact of clonal frequency on responses ^108, 109^, shows that after a period of arrest, T cells resume roaming within the lymphoid organ. Moreover, activated T cells leave the lymphoid organs to circulate and be recruited to tissues – especially if there are sites of inflammation. Further complexities related to inflammation and the kaleidoscope of the micro-environment include hypoxia, angiogenesis, and tumor-associated myeloid-lineage cells. The metabolic landscape differs among the arterial, venous, and lymphatic circulations as well as the tissues, which in turn will be different if challenged by metastatic cancer cells or microbial invasion (e.g. intracellular pathogens) ( Figure 4). Co-stimulation and hypoxia response mechanisms impact motility, mTOR activities, and T-cell phenotypes, although sometimes with opposite experimental results that may depend on the metabolic environment of the tested cells, the lymphocyte subset, whether the T cell had previously been activated, and the form of stimulus (e.g. cytokine) ^110– 115^. As a tumor grows, mutability can yield “self” peptides of altered sequence that may then be perceived as foreign (“non-self”) ^116^. Indeed, new evidence suggests a correlation or even mechanistic connection between the degree to which such epitopes are generated and the responsiveness to checkpoint inhibitors ^117, 118^. However, the universe of peptides that activate Treg may differ from those that engage TCR on conventional CD4 and CD8 T cells, and MHC-neopeptide complexes will include antagonists that drive clonal anergy ^119^.

## Imagining parts of a future

To recap, an attractive model based on “PI3K–AKT–mTOR” signals that drastically alter nuclear FoxO protein levels could link checkpoint therapies to changes in immune responsiveness controlled by Treg. However, constrained by the state of current technologies, the first-wave studies are based on relatively extreme loss- or gain-of-function changes that are fixed and static. In the worst instances, inferences are drawn and conclusions are strongly stated based on knockouts that yield starting T-cell populations identifiably different from the reference “wild-type controls”. But even setting aside this problem, temperance and caution are called for in making a dogma from reasonable inferences based on immutable perturbations in systems that have clear feedback inhibition and counter-regulation. Moreover, evidence is lacking as to where key changes in the relevant cells occur – with the cells in question highly dynamic. It is likely *a priori* that shifts in the environment as activated T cells move within and out from tissues modulate the probabilities of particular fates ( Figure 4).

Looking ahead, then, it is suggested that the list of needs includes the following:

1. Cultural change in science, with fewer oversimplifications that rely on overly categorical divisions between function of A versus function of B ( Figure 3). A central challenge stems from a combination of the overly definitive presentation of findings along with an implicit expectation of relatively universal “explanations” and an undue degree of “buy-in” to static conceptual schemata (explanatory cartoons).
2. More information on heterozygote phenotypes for loss-of-function perturbations of these pathways rather than focusing exclusively on the extremes – more broadly, better and more widespread attention to and precision with dose-response curves and understanding of stochastic variance ( Figure 2).
3. Development and use of “tools” and methodologies whereby graded changes in activity of the systems can be imposed – ideally, for more limited periods of time even than a laudable model making use of 4-OH-tamoxifen-activated estrogen receptor ligand-binding domains ^120^. Application of optogenetic tools or their marriage to new genetics may prove valuable in moving toward this goal, ideally with the added value of permitting spatially restricted activation ^121– 124^.
4. More ready supply of existing tools, along with development of better means for analyzing the actual pool of fuels (e.g. hexoses, glutamine, fatty acids, and oxygen) and metabolites in the interstitial spaces which are the soils among which cells are moving, along with
5. A capacity to follow the cells and better detect their signaling *in situ* ^124– 126^ across the timescales relevant to their development and functions.
